# Developing an Instrument for Assessing Self-Efficacy in Data Mining and Analysis

**DOI:** 10.3389/fpsyg.2020.614460

**Published:** 2021-01-15

**Authors:** Yu-Min Wang, Chei-Chang Chiou, Wen-Chang Wang, Chun-Jung Chen

**Affiliations:** ^1^Department of Information Management, National Chi Nan University, Puli, Taiwan; ^2^Department of Accounting, National Changhua University of Education, Changhua City, Taiwan; ^3^Department of Finance, National Changhua University of Education, Changhua City, Taiwan

**Keywords:** self-efficacy, data mining, measurement instrument, big data, artificial intelligence

## Abstract

With the continuous progress and penetration of automated data collection technology, enterprises and organizations are facing the problem of information overload. The demand for expertise in data mining and analysis is increasing. Self-efficacy is a pivotal construct that is significantly related to willingness and ability to perform a particular task. Thus, the objective of this study is to develop an instrument for assessing self-efficacy in data mining and analysis. An initial measurement list was developed based on the skills and abilities about executing data mining and analysis, and expert recommendations. A useful sample of 103 university students completed the online survey questionnaire. A 19-item four-factor model was extracted by exploratory factor analysis. Using the partial least squares-structural equation modeling technique (PLS-SEM), the model was cross-examined. The instrument showed satisfactory reliability and validity. The proposed instrument will be of value to researchers and practitioners in evaluating an individual’s abilities and readiness in executing data mining and analysis.

## Introduction

With the penetration and advent of data storage technologies and automatic data collection techniques, the big data age is coming. Although these technologies bring rich and diverse digital data to organizations, they can also cause serious information overload. Organizations of all sizes are under pressure to extract large amounts of data and process it into useful information and knowledge. Therefore, organizations increasingly need professionals to develop and deploy data mining technologies for competitive advantage (Nemati and Barko, 2003).

Data mining is a multi-disciplinary field (Chung and Gray, 1999; Feelders et al., 2000). Successful and effective data mining requires a collaborative effort in a number of areas, including statistics, artificial intelligence, database management, data visualization, subject area expertise, data analysis expertise, and data mining algorithms (Chung and Gray, 1999; Feelders et al., 2000; Nemati and Barko, 2003). However, at present instruments to properly and accurately measure individual abilities in data mining and analysis remain lacking. This study addresses this gap in research and practice.

Self-efficacy is an important construct in social science and information management (Compeau and Higgins, 1995). It has critical influences on task success and performance (Torkzadeh and Van Dyke, 2001). The purpose of this paper is to empirically develop an instrument for assessing an individual’s self-efficacy in data mining and analysis. Self-efficacy in data mining and analysis represents an individual’s judgment of their capabilities and skills to use data mining techniques for analysis and discovery in a given domain (Bandura, 1997; Wilson et al., 2007; Wang Y. Y. et al., 2019).

The remainder of this paper is organized as follows. Section “Background and Literature Review” reviews the related literature. Section “Research Methods” describes the research method and section “Results” presents the results of data analysis. Section “Application Analysis” describes the application analysis. Finally, the conclusion, implications, and research limitations are discussed in section “Conclusion and Implications.”

## Background and Literature Review

### Data Mining

In the past, corporate decisions were often made subjectively by decision makers, leading to errors. With the rapid development of science and technology, companies have gradually begun to use objective data to make decisions. In particular, the accumulation of data at large companies has increased rapidly and technology-assisted data analysis (e.g., data mining analysis) has gradually become an important tool for corporate decision-making. Data mining technology is an indispensable technology in the era of big data analysis. Hand et al. (2001) define data mining as the analysis of data sets (usually a large number of data sets) to identify unexpected relationships and summarize the data in novel patterns, and then provide useful information. Jain and Srivastava (2013) observed that data mining algorithms are divided into two functional types, predictive and descriptive, and eight application types, classification, estimation, forecasting, correlation analysis, sequence, time series, description, and visualization (Dunham, 2003).

Data mining technology is not only used in corporate decision-making, but is widely used in various industries. For example, in business management, Alola and Atsa’am (2020) applied data mining technology to measure the psychological capital of employees in the organization, and noted that when measuring the psychological capital of employees in recruitment interviews and promotion evaluations, data mining classification models can be useful as tools for human resource management. Zhen and Yao (2019) analyzed the lean production and technological innovation of the manufacturing industry based on the support vector machine algorithm and data mining technology. Data mining can discover novel, effective, potential, and finally understandable data patterns from a deeper level, and encode the data to predict the development trend of the enterprise. Machine learning support vector machine methods are used to analyze and model the collected data. Ding et al. (2019) indicated that the current cloud computing technology is developing rapidly, gradually integrating into IoT data mining technology and forming a new model. On this basis, the construction of an IoT data mining model based on cloud computing technology was studied. Another example is application in medicine. Zhao et al. (2020) used data mining to study the risk factors that can predict IHD during pheochromocytoma surgery, and observed that data mining techniques are increasingly being used in clinical and medical decision-making to provide continuous support for the diagnosis, treatment, and prevention of disease. Massi et al., 2020 noted that the healthcare industry is an interesting target for fraudsters. The availability of large amounts of data makes it possible to solve this problem through the use of data mining techniques, thereby making the review process more effective. The purpose of this research was to use the hospital discharge chart in the management database to develop a new type of data mining model specifically for fraud detection between hospitals. Qian and Liu (2020) proposed data mining technology that first determined the classification of index parameters. They then used this data mining technology to establish a sports training analysis mechanism to complete the construction of the index analysis model.

Data mining technology has also been widely used in the education field and is now being used more and more widely in teaching activities (Calders and Pechenizkiy, 2012; Maldonado and Seehusen, 2018). Data mining technology can be used to analyze educational data and explore educational research issues (Campagni et al., 2015). It can be used to improve educational practices and learning materials (Romero and Ventura, 2013), and to predict student performance, group students, plan courses, discover bad student behavior, model students, and classify courses based on student preferences (Romero and Ventura, 2010; Goyal and Vohra, 2012; Maldonado and Seehusen, 2018). The main focus of educational data exploration is to help solve problems related to the learning process of students, as well as to help schools conduct adaptive curriculum planning and students conduct adaptive learning (Calders and Pechenizkiy, 2012; Maldonado and Seehusen, 2018).

### Self-Efficacy

According to the theory of social cognition, perceptual self-efficacy is the key mechanism for exercising human agency within a causal structure involving the ternary causality of people, environment, and behavior (Bandura, 1986). Self-efficacy belief is an individual’s belief in their ability to achieve expected results, overcome obstacles, resist adversity, self-regulate in the face of urgent circumstances, discern many competing choices and negotiate important life changes (Basili et al., 2020). Self-efficacy means an individual’s confidence in their own problem solving and task completion ability (Sun and Chen, 2016; Ghazi et al., 2018). İncirkus and Nahcivan (2020) observe that self-efficacy refers to people’s belief in their ability to implement an action plan, deal with challenges, and make the judgments that make a particular action successful. Mamaril et al. (2016) and Liu et al. (2020) indicated that self-efficacy is an individual’s conjecture and judgment of whether they have the ability to complete a certain behavior, which can reflect the individual’s belief in taking appropriate action to address environmental challenges. It contains expectations of results and expectations of effectiveness (Bandura, 1997). The former is the belief that certain actions will ensure certain results, while the latter is the belief that one can complete these actions and obtain results (Sun and Chen, 2016). Bandura and Cervone (1986) and Sullivan et al. (2006) argue that since people who are confident in a task will expect success, concentrate on thinking about how to succeed, persist in facing difficulties, and avoid low self-efficiency tasks, self-efficacy beliefs are highly positively correlated with work and academic performance. Thus, when self-efficacy beliefs can be improved, performance improvement will occur (Dunlap, 2005; McLaughlin et al., 2008; Kuiper et al., 2010).

Many studies have explored the self-efficacy of students in academic fields and the self-efficacy of employees in practical fields. Research on employees largely explores personal self-efficacy in specific work situations (Bandura, 1986; Judge et al., 1998; Bandura and Locke, 2003). Bandura and Locke (2003) argue that self-efficacy is positively related to individual behavioral processes and results, such as perseverance in adversity, efforts to achieve high achievements, and ultimately high performance in various fields. Chae and Park (2020) indicate that expectations of personal self-efficacy determine how much task-related effort will be expended. Therefore, beliefs related to self-efficacy are the most powerful predictors of individual behavior and persistence in adversity (Bandura, 1986). Bandura (1986) and Bandura and Locke (2003) contend that when individuals have a high sense of self-efficacy, the resources they are willing to invest in tasks will increase, leading to better results. Other studies have explored the relationship between self-efficacy and entrepreneurial enthusiasm and entrepreneurial behavior (Shane et al., 2003; Murnieks et al., 2014). Shane et al. (2003) observed that self-efficacy and enthusiasm are two important factors in maintaining entrepreneurial efforts. Sun (2020) showed that self-efficacy mediates the relationship between entrepreneurial enthusiasm and entrepreneurial behavior. Researchers have also explored general self-efficacy, individuals’ perception of their ability to perform in various situations, in the general workplace (Smith, 1989; Scholz et al., 2002; Chen et al., 2004). Results show that general self-efficacy is positively correlated with job performance (Beattie et al., 2016) and knowledge sharing (Srivastava et al., 2006). Chae and Park (2020) explored the relationship between an employee’s general self-efficacy and task performance and knowledge-sharing. The results showed that the high general self-efficacy of key employees has a positive impact on task performance but has a negative impact on knowledge sharing.

Most studies of the self-efficacy of students agree that self-efficacy has a positive impact on learners’ academic achievement and personal success (Vancouver et al., 2001; Honicke and Broadbent, 2016; Basili et al., 2020). Fernandez-Rio et al. (2017) indicated that academic self-efficacy beliefs affect the perception of ability in the self-regulation process that is beneficial to learning. Cooper (2015) demonstrated that self-efficacy can help students at risk overcome their at-risk conditions and positively impact their academic performance. Schunk (1994) and Carroll et al. (2009) demonstrated that students with higher self-efficacy beliefs can better manage their own learning and are more likely to do better academically. Klassen and Usher (2010) and Talsmaa et al. (2018) all observed that people with high self-efficacy set more difficult goals, put in more effort, persist in challenges for a longer time, and show resilience in adversity, which can improve academic achievement (Bandura, 1997). Klassen and Usher (2010) contended that self-efficacy has a key and powerful influence on academic achievement. Pajares and Kranzler (1995) found that self-efficacy can effectively predict academic achievement. Multon et al. (1991), Richardson et al. (2012), and Honicke and Broadbent (2016) conducted a meta-analysis of self-efficacy, finding that self-efficacy is strongly correlated with academic achievement.

Many researchers have found that self-efficacy plays an important role in the process and results of individual behavior. However, since self-efficacy is a kind of behavioral cognition, a psychological scale to measure personal self-efficacy is needed. A number of different self-efficacy scales have been developed for various fields, such as self-efficacy in the medical field (Lorig et al., 1989; İncirkus and Nahcivan, 2020), general self-efficacy scales in the workplace (Chen et al., 2004), self-efficacy scale for engineering education (Mamaril et al., 2016), multi-dimensional self-efficacy scale for adolescents (Bandura, 1990), teacher research self-efficacy scale (Wester et al., 2019), teacher self-efficacy scale for student-oriented teaching (Kilday et al., 2016), college student self-efficacy scale (Khasawneh et al., 2009), and a mathematical self-efficacy energy scale (Betz and Hackett, 1983). Based on the development of education in the high-tech era, the popularization of technology-assisted teaching has led many researchers to study the role of self-efficacy when the Internet or technology is applied to teaching, and develop numerous Internet and technology-related self-efficacy scales, such as the Internet self-efficacy scale (Hsu and Chiu, 2004; Kao et al., 2011), the computer ethical self-efficacy scale (Kuo and Hsu, 2001), and the Internet ethical self-efficacy scale (Williamson et al., 2011). With the development of Internet and high technology, though big data analysis and artificial intelligence have gradually become common across various industries, data mining and artificial intelligence self-efficacy scales remain lacking. Therefore, the main purpose of this research is to develop a self-efficacy scale for data mining and analysis.

## Research Methods

Based on the prior measures and definitions of self-efficacy, this study conceptually defines “self-efficacy in data mining and analysis” as an individual’s judgment of his or her ability to successfully execute data mining and analysis. The initial instrument, which consisted of 28 items, was developed based on the review of the literature on skills and abilities for executing data mining and analysis (Fayyad et al., 1996; Chung and Gray, 1999; Mitchell, 1999; Chapman et al., 2000; Feelders et al., 2000; Liao, 2008; Han et al., 2011; Tufféry, 2011; McCormick et al., 2013; Singhal and Jena, 2013; Abbott, 2014; Jian and Hsu, 2014; Xue, 2014; Marvin, 2016; Salcedo and McCormick, 2017; Struhl, 2017; Chang and Kung, 2019; Liao and Wen, 2019; Wang, 2019; Wang Y. S. et al., 2019) and expert experience. Three global items for measuring perceived overall self-efficacy were added to serve as a criterion. All items were measured using a seven-point Likert-type scale with anchors of “(1) strongly disagree, (2) disagree, (3) slightly disagree, (4) neutral, (5) slightly agree, (6) agree, and (7) strongly agree.” Table 1 shows all 31 items.

**TABLE 1:** The measurement items.

**Items**
Q1. I clearly understand the main applications of data mining, e.g., classification, estimation, forecasting, association, and cluster analysis
Q2. I clearly understand the procedure and main steps of data mining
Q3. I am familiar with standards for data mining and modeling
Q4. I have the ability to conduct data mining in a professional field (such as consumer behavior analysis, sales data) to discover useful information or knowledge
Q5. I have the ability to understand and interpret the outputs derived from data mining
Q6. I am familiar with at least one major programming language for data mining, such as R, Python, or Java
Q7. I think I have the programming skills required for data mining
Q8. I know how to use information retrieval methods to find useful information from a large amount of data
Q9. When I search for information, I can use keyword search accurately
Q10. I have the relevant ability of database system
Q11. I have the ability to clean, select, transform, and synthesize data
Q12. I have the ability to execute online analytical processing (OLAP)
Q13. I have the ability to use SQL (Structured Query Language)
Q14. I have the ability to build a data warehouse
Q15. I am familiar with at least one data exploration tool, such as WEKA, RapidMiner, IBM SPSS modeler, and Statistica
Q16. I have the ability to carry out pre-processing of data mining
Q17. I have the ability to execute classification analysis
Q18. I have the ability to execute cluster analysis
Q19. I have the ability to execute the feature selection
Q20. I have the ability to visualize the data
Q21. I have the relevant statistical skills required for data mining
Q22. I have the ability to execute the decision tree analysis
Q23. I have the ability to execute discriminant analysis
Q24. I have the ability to execute association analysis
Q25. I have the ability to execute sequential pattern analysis or causal analysis
Q26. I have the ability to execute time-series analysis
Q27. I have the ability to execute artificial neural networks (ANN) analysis
Q28. I have the ability to use at least one data mining technique for data analysis or discovery
G1. Overall, I think I have professional ability in data mining*
G2. Overall, I think my data mining skills capabilities meet the needs of practitioners*
G3. Overall, I think I have good and complete data mining knowledge*

*Criterion item.

The survey methodology was adopted and empirical data for this study were collected using an Internet questionnaire survey in Taiwan. University students with data mining knowledge or experiences were qualified to participate in the survey, and were asked to fill in the questionnaire based on their experiences and self-perceptions. Every respondent in the survey was given an NT 100-dollar coupon as an incentive. The survey duration was 2 months: from April to May in 2020. This study obtained 103 useful responses. There were more females than males in the sample (51.5 and 48.5%). The proportion of college students in the sample is higher than that of graduate students (85.4 and 14.6%). The respondents had an average age of 21.6 years. On average, they took 4.03 courses and 12.57 credits in data mining.

Data from 103 university students was tested against the proposed 28-item instrument using a two-step assessment approach. In the first stage, the exploratory factor analysis (EFA) and the criterion-related analysis was used to purify the measure, remove noise items, and acquire factor structure. In the second stage, the partial least squares-structural equation modeling (PLS-SEM) was used to assess the hierarchical component model (HCM) based on the EFA result. Internal consistency (reliability), convergent validity, and discriminant validity were checked for the model.

## Results

### EFA Results

Exploratory factor analysis was used to purify the measurement instrument. Before conducting the EFA, three tests were performed to check the adequacy of the survey data for EFA. First, Cronbach’s α coefficient was computed to ensure the internal inconsistency of the measurement items (Churchill, 1979). The results showed that the 28-item instrument had an α coefficient of 0.97, indicating that the measure was unidimensional. Second, Bartlett’s test of sphericity was used to assess the overall significance of the correlations among the measurement items (Hair et al., 1998). The results demonstrated a satisfactory suitability of the data for factor analysis (χ^2^ = 3387.31, *p* < 0.001). Third, the Kaiser–Meyer–Olkin statistic was computed for checking sampling adequacy. The statistical score was 0.91 and greater than 0.50, indicating high shared-variance and relatively low uniqueness (Hair et al., 1998). These test results suggested that EFA was worth pursuing.

The principle-components analysis was used as an extraction technique and varimax method was used to rotate the factor matrix. Referring to Kaiser (1960), Sethi and King (1991), and Hair et al. (1998), four rules were applied in EFA: (1) a factor with an eigenvalue greater than 1.00 was retained; (2) an item with all factor loadings below 0.55 was removed; (3) an item with two or more factor loadings (rounding numbers) above 0.55 was dropped; and (4) an item with two or more correlation coefficients with other items greater than 0.85 was removed. Table 2 shows the EFA results. The results show that 77.54 percent of variance is explained by four factors and 19 items are left in the instrument. These factors are labeled “Data mining techniques,” “Programming and database,” “Basic knowledge and procedure of data mining,” and “Data retrieval and statistical presentation.” The respective Cronbach’s α coefficients are 0.94, 0.91, 0.87, and 0.84. All the coefficients exceed the acceptable standard of 0.70.

**TABLE 2:** EFA results.

**Items**	**Factor 1**	**Factor 2**	**Factor 3**	**Factor 4**
Q1			0.56	
Q2			0.83	
Q3			0.61	
Q4			0.78	
Q6		0.86		
Q7		0.85		
Q8				0.59
Q9				0.68
Q10		0.74		
Q12	0.67			
Q13		0.82		
Q15	0.69			
Q16	0.70			
Q20				0.78
Q21				0.69
Q22	0.84			
Q24	0.78			
Q26	0.83			
Q27	0.82			
Eigenvalue	5.40	3.57	2.97	2.79
Variance explained	28.44%	18.80%	15.65%	14.66%
Cumulative variance explained	28.44%	47.23%	62.88%	77.54%
α coefficient	0.94	0.91	0.87	0.84

*Factor 1, data mining techniques; Factor 2, programming and database; Factor 3, basic knowledge and procedure of data mining; Factor 4, data retrieval and statistical presentation.*

The criterion-related validity was assessed by the correlation between the sum of scores on all 19 items in the instrument and the validity criterion (sum of three criterion items). The correlation was 0.78, significant at 0.001, representing satisfactory criterion-related validity.

The multitrait-multimethod (MTMM) approach was used for evaluating the convergent and discriminant validity of the instrument. Table 3 shows the correlation coefficients between items. Convergent validity is acceptable when the correlation coefficients of the same factor are significantly different from zero and large enough for further investigation (Doll and Torkzadeh, 1988). The smallest within-factor correlation coefficients are: Data mining techniques = 0.50, Programming and database = 0.60, Basic knowledge and procedure of data mining = 0.43, Data retrieval and statistical presentation = 0.54. All coefficients are significantly different from 0 (*p* < 0.01) and large enough, demonstrating the convergent validity of the measures.

**TABLE 3:** Correlation coefficient between items.

	**Q1**	**Q2**	**Q3**	**Q4**	**Q6**	**Q7**	**Q10**	**Q13**	**Q8**	**Q9**	**Q20**	**Q21**	**Q12**	**Q15**	**Q16**	**Q22**	**Q24**	**Q26**
Q2	**0.66**																	
Q3	**0.63**	**0.72**																
Q4	**0.50**	**0.67**	**0.57**															
Q6	0.41	0.47	0.49	0.46														
Q7	0.30	0.35	0.43	0.41	**0.80**													
Q10	0.35	0.51	0.47	0.50	**0.71**	**0.60**												
Q13	0.43	0.42	0.57	0.35	**0.80**	**0.70**	**0.76**											
Q8	0.60	0.50	0.53	0.48	0.58	0.52	0.50	0.46										
Q9	0.39	0.44	0.34	0.50	0.48	0.36	0.59	0.38	**0.69**									
Q20	0.60	0.39	0.43	0.46	0.42	0.35	0.47	0.46	**0.64**	**0.53**								
Q21	0.48	0.24	0.37	0.21	0.27	0.20	0.32	0.31	0.51	**0.43**	**0.63**							
Q12	0.47	0.65	0.65	0.49	0.53	0.46	0.50	0.52	0.56	0.38	0.36	0.35						
Q15	0.49	0.60	0.69	0.45	0.54	0.44	0.39	0.45	0.50	0.26	0.28	0.35	**0.77**					
Q16	0.58	0.49	0.55	0.32	0.56	0.47	0.36	0.51	0.61	0.36	0.57	0.56	**0.64**	**0.72**				
Q22	0.48	0.53	0.63	0.29	0.38	0.31	0.35	0.46	0.56	0.36	0.41	0.56	**0.68**	**0.60**	**0.65**			
Q24	0.51	0.39	0.58	0.34	0.43	0.37	0.43	0.49	0.58	0.31	0.51	0.68	**0.62**	**0.54**	**0.61**	**0.78**		
Q26	0.49	0.55	0.63	0.37	0.39	0.36	0.39	0.42	0.65	0.42	0.52	0.61	**0.71**	**0.62**	**0.68**	**0.84**	**0.78**	
Q27	0.48	0.47	0.63	0.32	0.43	0.45	0.39	0.46	0.65	0.37	0.45	0.53	**0.71**	**0.66**	**0.67**	**0.75**	**0.72**	**0.84**

*The correlation coefficients between items of the same factor will be shown in bold.*

The discriminant validity for each item was assessed by counting the number of times correlated more closely with items of other factors than items of its own theoretical factor (Wu and Wang, 2006). Such counts should be less than 50 percent of the comparisons. As shown in Table 3, there were 45 violations out of 264 comparisons, representing acceptable discriminant validity.

### PLS-SEM Results

**FIGURE 1:**
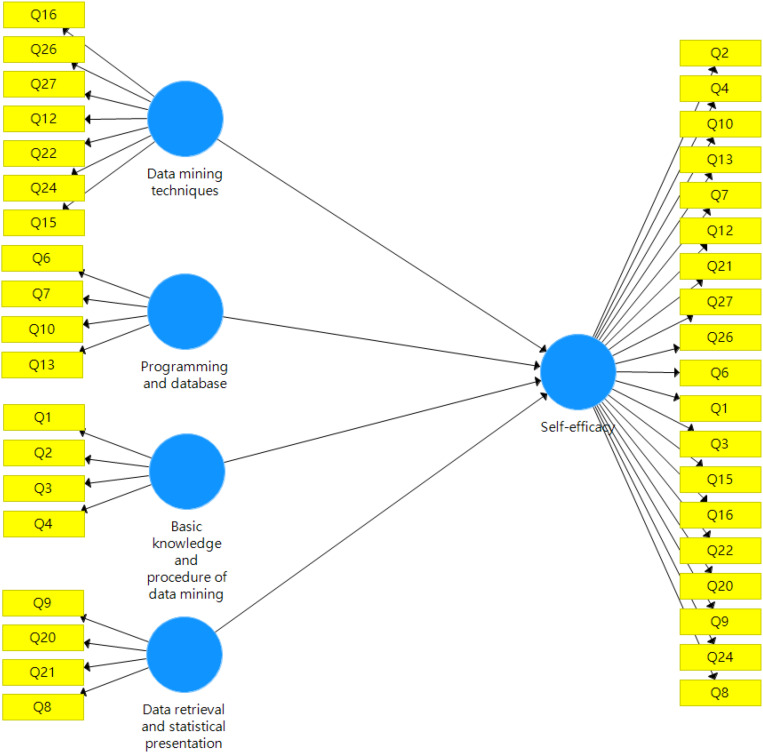
The higher-order measurement model.

According to the two-stage HCM method suggested by Hair et al. (2017) and the rationale of EFA results, a reflective-formative measurement model was built. The repeated indicators approach was adopted for analyzing the higher-order measurement model (Figure 1). This model hypothesized that the four reflective first-order factors formed one second-order factor. Self-efficacy in data mining and analysis is multi-faceted and the four factors of Data mining techniques, Programming and database, Basic knowledge and procedure of data mining, and Data retrieval and statistical presentation are components of self-efficacy in data mining and analysis. Therefore, the formative type (components second-order construct) is reasonable. The 19 items are reflective indicators of these four first-order factors.

There are two parts in the measurement evaluation. First, internal consistency (rho_A), convergent validity (AVE, outer loading) and discriminant validity (HTMT) were checked for the reflective part of the model, the measurement of the four factors. Second, the convergent validity, collinearity, and significance of the path coefficients were evaluated for the formative part of the model, the four factors forming the higher-order component, self-efficacy.

Table 4 shows the PLS results and relative standards of the reflective part of the measurement model. All rho_A values for the factors exceeded the recommended value of 0.7, supporting internal consistency. The average variance extracted (AVE) values for the four factors are 0.74, 0.80, 0.72, and 0.68. All AVE values are greater than 0.5, justifying the convergent validity. As shown in Table 4, the outer loadings of all items are significant and above 0.7, confirming the convergent validity of this measure. Finally, the heterotrait-monotrait (HTMT) was used to assess discriminant validity. As shown in Table 4, all HTMT values are below the threshold value of 0.9, confirming discriminant validity (Hair et al., 2017). In sum, the reflective part of the measurement model demonstrates adequate reliability and validity.

**TABLE 4:** PLS results: The reflective part.

**Tests**	**Factor 1**	**Factor 2**	**Factor 3**	**Factor 4**
rho_A	0.94	0.92	0.88	0.86
	All coefficients are above the minimum standard of 0.7			

AVE	0.74	0.80	0.72	0.68

	All AVEs are above the minimum standard of 0.5			

Outer loading	0.81–0.92	0.86–0.93	0.79–0.90	0.77–0.88
	All loadings are above the minimum standard of 0.7			

HTMT	0.61–0.76	0.61–0.64	0.64–0.76	0.65–0.75
	All HTMT indexes are below the maximum threshold of 0.9			

*Factor 1, data mining techniques; Factor 2, programming and database; Factor 3, basic knowledge and procedure of data mining; Factor 4, data retrieval and statistical presentation.*

Table 5 shows the PLS results and relative standards of the formative part of the measurement model. Three analyses were executed. First, convergent validity was evaluated. Convergent validity is the extent to which a measure correlates positively with other measures of the same construct using different indicators (Hair et al., 2017). Therefore, this study used redundancy analysis for assessing convergent validity. The redundancy analysis method is useful for analyzing a directional relationship between two sets of multivariate data (Lambert et al., 1988). We created one exogenous self-efficacy construct that are measured by 19 items and one endogenous self-efficacy construct that are first measured by three global items. Then we examine the path coefficient through which the exogenous construct influences the endogenous construct. The path coefficient is 0.82, above threshold value of 0.8, confirming convergent validity (Wong, 2019). Second, the collinearity issue was assessed. Collinearity should be evaluated in a model with multiple variables as a possible predictor-predictor redundancy phenomenon (Kock and Lynn, 2012). When two or more predictor variables in a multiple regression model are highly correlated, multicollinearity occurs, which will cause the variance inflation and increase the type I error, making some coefficients appear significant when they are not (Lombardi et al., 2017). When the variance inflation factor (VIF) is higher than the threshold value of 5.0, a potential collinearity problem can exist. As shown in Table 5, all VIF values are below 5.0, indicating no collinearity problem. Third, the significance of the path coefficients from the four factors to the high-order self-efficacy construct was examined. The path coefficients are 0.51, 0.21, 0.22, and 0.22. All path coefficients are significant.

**TABLE 5:** PLS results: The formative part.

**Tests**	**Results**
Convergent validity (redundancy analysis)	Path coefficient = 0.82 The path coefficient (HOC → criterion) is above the minimum standard of 0.8
Collinearity	VIF = 2.47, 1.73, 2.25, 2.16 All VIFs are below the maximum threshold of 5.0
Significance of path coefficients	Path coefficients = 0.51, 0.21, 0.22, 0.22 All path coefficients (LOC → HOC) are significant at 0.001 level

All indices and statistics in Tables 4, 5 have reached relevant assessment standards. The measurement model has satisfactory reliability and validity.

## Application Analysis

Through rigorous empirical analysis, this study has developed a reliable and valid instrument for measuring an individual’s self-efficacy in data mining and analysis. This section presents the application analysis of the instrument from three perspectives. First, the correlation between education and self-efficacy in data mining and analysis is assessed. Second, measurement invariance from the gender perspective is evaluated. Finally, the norms of this instrument are developed.

### The Correlation Between Education and Self-Efficacy in Data Mining and Analysis

This study found that there is a significant positive correlation between total self-efficacy level and credits taken by university students in data mining and analysis related courses. The correlation coefficient is 0.41, significant at 0.001. This relationship is significant and positive. The regression analysis is also tested. The independent variable is credits taken by university students in data mining and analysis related courses, and the dependent variable is total self-efficacy level. The results are β = 0.41, *T* = 4.57, and significance level < 0.001. These findings support the effectiveness of university education in the data mining and analysis domain.

### Measurement Invariance

Measure invariance is also called measurement equivalence (Wong, 2019). It refers to the degree of a measure retains the measurement properties across observations and contexts (Mangos and Johnston, 2008). Measure invariance should be checked prior to executing multi-group analysis in the future study. This study assessed the measurement invariance from the gender perspective. Referring to Hair et al. (2017) and Wong (2019), three steps were applied: (1) Configural invariance is developed using the same path model, data treatment, and analysis algorithm. (2) Compositional invariance is evaluated by comparing path coefficients. (3) Composite means and variances are assessed if compositional invariance exists.

For analysis, we split the sample into two groups based on gender. The male group has 53 responses and the female group has 50 responses. First, the same two PLS path models for these two groups were developed. The analysis parameters and algorithm were set the same for configural invariance. Then path coefficients were estimated and compared for examining compositional invariance. The modified two independent-sample *t*-test of Keil et al. (2000) was used to compare whether the path coefficients between male and female samples are significantly different. The results are shown in Table 6. One relationship (Data mining techniques → Self-efficacy) was found to have different path coefficients. This implies that males and females have different perceptions about the influence of data mining techniques on self-efficacy. Compositional variance in measuring data mining techniques may exist across gender.

**TABLE 6:** Comparisons of path coefficients by gender.

**Paths**	**Male**		**Female**		***P*-value**
	**β**	**SD**	**β**	**SD**	
Data mining techniques → Self-efficacy	0.54	0.04	0.43	0.02	0.03
Programming and database → Self-efficacy	0.22	0.04	0.23	0.02	0.86
Basic knowledge and procedure of data mining → Self-efficacy	0.21	0.03	0.25	0.02	0.27
Data retrieval and statistical presentation → Self-efficacy	0.24	0.02	0.21	0.02	0.16

### Norms

The composite scores were computed by summing the 19- item scores. However, a raw composite score on a measurement instrument may be not sufficiently informative (Churchill, 1979). A better way of assessing an individual’s self-efficacy is to compare the individual score with norms – the total distribution of the scores achieved by other people. The tentative norm of the self-efficacy instrument was presented in Table 7. These statistics offer a frame of reference and comparison for potential instrument users. The instrument users can use the norms as the benchmark for evaluating relative abilities and scores against others.

**TABLE 7:** Percentile scores for the instrument.

**Percentile**	**Composite score**				
	**Total**	**Factor 1**	**Factor 2**	**Factor 3**	**Factor 4**
10	45.40	10.80	8.00	9.40	12.00
20	57.00	14.00	11.00	12.00	13.00
30	61.20	18.00	15.00	14.00	15.00
40	68.20	21.00	16.00	15.00	16.00
50	74.00	23.00	19.00	16.00	17.00
60	77.80	27.40	20.00	17.00	19.00
70	87.60	29.00	21.00	19.00	19.00
80	94.00	31.20	23.00	20.00	20.20
90	99.60	35.00	24.00	21.00	22.60

*Factor 1, data mining techniques; Factor 2, programming and database; Factor 3, basic knowledge and procedure of data mining; Factor 4, data retrieval and statistical presentation.*

## Conclusion and Implications

Most data-mining studies focus on development of innovative algorithms, comparisons of different algorithms, and application analysis. However, relatively few studies evaluate individuals’ capabilities and talents in data mining. This study is a pioneering effort to develop and validate an instrument for assessing an individual’s self-efficacy in data mining and analysis. The measure items are developed based on relevant data-mining literature and practical experiences. The instrument is purified and validated empirically. Finally, nineteen items are exclusively used to assess an individual’s self-efficacy in data mining and analysis. The results reveal that self-efficacy in data mining and analysis is a higher-order construct composed of four dimensions: Data mining techniques, Programming and database, Basic knowledge and procedure of data mining, and Data retrieval and statistical presentation. The results enhance our understanding of the nature and dimensionality of self-efficacy in data mining and analysis. The research findings have several implications for practitioners and researchers.

First, the instrument developed in this study can be used as an assessment and diagnosis tool. Students and practitioners can use this instrument to assess their abilities in data mining and analysis and take action to address weaknesses. Enterprises can use this instrument to assess employee abilities. When enterprises recruit data-mining professionals, they can design exam questions using the four dimensions. Instructors in universities can refer to the items, dimensions, and relative influences of these dimensions in designing data-mining programs and allocating course credits.

Second, this study finds that “data mining techniques” have the highest influence on self-efficacy (β = 0.51) among the four factors. This implies that “data mining techniques” are the requisite capabilities that individuals need to effectively perform data mining and analysis. When individuals have mastery of data mining techniques, they have the knowledge and abilities to handle decision tree, association, time-series, and artificial neural network analysis, and the pre-processing of data mining. These are indispensable and fundamental capabilities.

Third, this study also finds that the other three factors have significant and similar influences (β coefficients are between 0.21 and 0.22). This finding supports the claim that data mining is a multi-disciplinary field (Chung and Gray, 1999; Feelders et al., 2000). Since executing data mining requires cross-domain knowledge and skills, individuals should possess more than basic data mining techniques. If they want to successfully execute data mining projects and obtain correct outcomes, expertise such as programming and database use, basic knowledge and procedure of data mining, and data retrieval and statistical presentation, should be possessed.

Fourth, this study finds that education and self-efficacy are positively correlated. This implies that the higher the number of credits related to data mining, the higher the self-efficacy. This not only supports the effectiveness of university education, but also encourages students who want to have the abilities in data mining and analysis to take more relevant courses.

Finally, measure variance in the “data mining techniques” dimension may exist across genders. This issue should be re-verified with more samples. If measure variance remains, researchers should address gender difference in the influence of data mining techniques on self-efficacy.

This research has several limitations. First, this research only takes students as the survey object for analysis. However, data mining and analysis are applied in practical domains. It is thus possible that people who work in practical applications of data mining technology will have different self-efficacy. In the future, people working in practical applications of data mining should be surveyed for further analysis. Second, the sample size of the research is not large and the sample does not include students of diverse backgrounds. Future research should expand coverage to students from different backgrounds and compare the differences among them in self-efficacy of data mining and analysis.

## Data Availability Statement

The datasets presented in this article are not readily available because when collecting the survey data, we had a promise to the respondents that the response contents would not be disclosed and be given to the third parties. Requests to access the datasets should be directed to corresponding author.

## Ethics Statement

Ethical review and approval was not required for the study on human participants in accordance with the Local Legislation and Institutional Requirements. Written informed consent from the participants was not required to participate in this study in accordance with the National Legislation and the Institutional Requirements. However, consent was implied via completion of the questionnaire.

## Author Contributions

Y-MW contributed to the research topic, data collection, statistical analysis, developing implications, and writing. C-CC took charge in literature review, writing the manuscript, and responsible for correspondence. W-CW developed the instrument and designed the questionnaire. C-JC contributed to data collection and practical implications. All authors contributed to the article and approved the submitted version.

## Conflict of Interest

The authors declare that the research was conducted in the absence of any commercial or financial relationships that could be construed as a potential conflict of interest.
